# Closing the counselling gap: a qualitative study to strengthen rights-based family planning in Pakistan

**DOI:** 10.1136/bmjopen-2025-116059

**Published:** 2026-08-24

**Authors:** Syed Khurram Azmat, Muhammad Ali Awan, Ellen Mpangananji Thom, Qudsia Uzma, Imran Sheikh, Grace Kapustianyk, James Kiarie, Moazzam Ali

**Affiliations:** 1 APPNA - Institute of Public Health, Jinnah Sindh Medical University, Karachi, Pakistan; 2 Monitoring, Evaluation, Research and Learning, Integrity Global, London, UK; 3 School of Nursing, Midwifery and Health Systems, University College Dublin, Dublin, Ireland; 4 World Health Organization Country Office for Pakistan, Islamabad, Pakistan; 5 Experts International, Karachi, Pakistan; 6 Life course, Health and Reproduction, World Health Organization, Geneva, Switzerland

**Keywords:** Health Services, REPRODUCTIVE MEDICINE, QUALITATIVE RESEARCH, Quality in health care

## Abstract

**Objective:**

To identify client and provider perspectives on contraceptive counselling in Pakistan and inform the development of an effective, rights-based counselling intervention package.

**Design:**

A formative, multimethod qualitative study was conducted using in-depth interviews, non-participant observations of counselling encounters and thematic analysis. The study was embedded within a WHO-led multiphase complex intervention.

**Setting:**

Primary care-level family planning facilities (public and private) in the urban and peri-urban districts of Islamabad and Rawalpindi.

**Participants:**

The analysis reported in this paper includes 72 married family planning clients (36 women and 36 men), 15 frontline healthcare providers and 72 observed counselling sessions across three facilities. Broader stakeholder interviews with programme managers, public-sector officials and donor/development partners were collected for the wider multiphase study but are not analysed in this paper.

**Results:**

Three overarching themes were identified: (1) counselling was often understood as basic method-related information rather than a structured, rights-based decision-making process; (2) public and private facilities differed in counselling depth, method choice, privacy and use of visual/couple-centred approaches; and (3) system constraints, including time pressure, commodity limitations, limited follow-up mechanisms, weak privacy arrangements and inconsistent training, shaped the quality of counselling. Observation data supported interview findings by showing variation in privacy, side-effect discussion, client engagement and follow-up advice across facilities.

**Conclusions:**

Findings from participating facilities suggest that the current counselling model in these settings inadequately supports informed, autonomous reproductive choices. A redesigned, context-sensitive counselling package is needed to address these gaps. This should be supported by improved training, supportive supervision, inclusive tools, systemic reforms and enhanced follow-up mechanisms that are essential to increase decision-making autonomy and contraceptive continuation.

**Trial registration number:**

NCT06081842.

**STRENGTHS AND LIMITATIONS OF THIS STUDY**

- This study used participatory methods, ensuring stakeholder voices guided intervention design.
- It examined both client and provider perspectives across public and private sectors.
- Findings are grounded in real-world service environments.
- Transferability may be limited beyond comparable urban and periurban primary care settings because the study was conducted in three facilities in Islamabad/Rawalpindi.
- Observational checklists may not fully capture subtle communication dynamics.

## Introduction

Access to family planning (FP) is widely recognised as a fundamental human right. Yet globally more than 218 million women have an unmet need for contraception, with the largest burden concentrated in low- and middle-income countries (LMICs).^1 2^ Counselling is a cornerstone of FP service delivery but often fails to support informed decision-making and client autonomy. Although modern contraceptive methods are increasingly available, Pakistan continues to face persistently high levels of unmet need and method discontinuation, frequently linked to ineffective, incomplete or coercive counselling practices.^3^

Despite having one of the oldest FP programmes in South Asia, Pakistan has yet to meet its target indicators. The Pakistan Demographic and Health Survey 2017–2018 reported that only one in three married women uses any contraceptive method, with modern method use substantially lower.^4^ More recent national analyses estimate that 8.6 million women in Pakistan continue to face an unmet need, and nearly half of all pregnancies are unintended, with profound implications for maternal morbidity, mortality and gender equity.^3^

Quality-of-care frameworks identify method choice, information provision, interpersonal relations, technical competence, follow-up and continuity as core elements of FP service quality.^5 6^ Person-centred FP approaches further emphasise dignity, autonomy, privacy, communication, trust and respectful care as central to quality, while recent intervention work highlights the need to strengthen counselling to improve client decision-making, autonomy and contraceptive need fulfilment^7 8^ and literature demonstrates that quality of contraceptive counselling strongly influences contraceptive uptake, continuation and protection of reproductive autonomy.^7^ The WHO explicitly frames contraceptive information and services as human rights obligations, emphasising non-discrimination, informed choice, privacy, accountability and freedom from coercion.^8^ Building on this foundation, recent conceptual work has advanced rights-based frameworks of ‘contraceptive agency’ and developed person-centred measures, such as the Quality of Contraceptive Counselling scale, which foregrounds respect, dignity, shared decision-making and support for clients’ reproductive goals.^9^ Therefore, in Pakistan, the need for formative evidence is particularly important because recent studies continue to identify gaps in counselling quality, provider capacity, method choice, service consistency and client-centred delivery across routine service platforms.^8 10 11^

In Pakistan, public-sector FP services are intended to provide contraceptive information and selected methods through government health facilities and community-based workers, although availability, privacy, counselling quality and follow-up vary across sites.^4 11^ Private and non-governmental providers may offer additional opportunities for FP access, service delivery innovation and client-centred counselling support, but access can still be shaped by cost, location, service availability and clients’ ability to navigate providers.^10 12^ These public–private differences are therefore important for understanding how counselling quality is produced in routine practice, especially when counselling is expected to support informed choice, respectful care, method continuation and reproductive autonomy.^5–8^

Despite this normative consensus, no globally agreed operational definition of ‘quality contraceptive counselling’ exists, particularly for routine public-sector settings in LMICs. An expert think tank convened by WHO proposed a working definition centred on eliciting clients’ fertility intentions and preferences, neutrally presenting a broad method mix, ensuring informed and voluntary choice, and providing structured follow-up; however, substantial evidence gaps remain on implementation and measurement in real-world programmes.^13 14^ Systematic reviews further show that although enhanced counselling may improve knowledge and short-term uptake, effects on continuation and autonomy are inconsistent, and many studies lack an explicit rights-based lens.^15^ Newer approaches, such as Balanced Counselling Strategy Plus (BCS+), the Rapport–Explore–Decide–Implement Counselling Framework and Counselling for Choice (C4C), aim to structure counselling around client priorities using simple job aids. Yet, evidence on their effectiveness in South Asian public-sector contexts remains limited.^16^

The Pakistan context illustrates these challenges vividly. National surveys show high awareness but fragile contraceptive use: almost all women can name at least one modern method, yet modern contraceptive prevalence remains close to one quarter, with wide socio-economic and geographical disparities.^3 4^ Contraceptive discontinuation is common, with side-effects and health concerns consistently emerging as leading reasons for stopping methods across national and community-based studies.^17^ Evidence from Pakistan and comparable settings indicates that better interpersonal quality of care, including respectful and comprehensive counselling, is associated with lower discontinuation and higher client satisfaction. Yet, such quality remains uneven and poorly monitored.

Within Pakistan’s FP programmes, counselling has historically been framed instrumentally, as a means to increase uptake or meet numeric targets, rather than as a process to support informed, person-centred decision-making. Training curricula reference the GATHER Family Plannig Counselling Framework *(GATHER is a six-component client-centred family planning counselling approach - **G**reet, **A**sk, **T**ell, **H**elp, and **R**eturn/**R**efer, used to support informed family planning choice, appropriate method use, and follow-up)* the BCS and other job-aid-based approaches. Still, these are often delivered in didactic formats without sufficient adaptation to the realities of high patient volumes, stock-outs, workforce shortages and gendered power relations in consultations.^3^ Emerging national assessments indicate fragmented implementation of rights-based guidance, weak supervision and limited use of provider checklists or client-experience indicators, particularly in public-sector facilities.^3^ There is also little empirical work from Pakistan that systematically triangulates client, provider and policymaker perspectives to assess how counselling is practised across the public and private sectors and how closely it aligns with global rights-based standards.

This study contributes to the literature by: (1) operationalising WHO’s rights-based counselling framework^18^ in a low-resource, dual-sector (public/private) setting; (2) triangulating client in-depth interviews (IDIs), provider IDIs and non-participant observations^1^ of counselling sessions; (3) purposively including equal numbers of male and female clients to examine gendered dynamics; and (4) embedding findings within a pre-specified, multiphase complex intervention (theory-driven, with a subsequent cluster RCT - Randomised Control Tril). Accordingly, this study responds to the identified gaps through a formative, participatory qualitative phase embedded within a WHO-led, multiphase complex intervention project on contraceptive counselling in Pakistan.^8^ Grounded in WHO’s human-rights framework for contraceptive information and services^19^ and recent global consensus work on defining quality counselling,^13 14^ the project is situated in primary care-level facilities in Islamabad and Rawalpindi, where most clients first encounter FP services. The formative phase aimed to:

1. Explore how clients, providers and key stakeholders conceptualise and experience contraceptive counselling in routine practice.
2. Identify interpersonal, organisational and system-level barriers to rights-based, client-centred counselling, including those contributing to method discontinuation and perceived coercion.
3. Generate context-specific insights to inform the co-design of a pragmatic counselling package to be evaluated in a subsequent cluster randomised trial. By documenting these perspectives and practice gaps, this paper contributes to the global evidence base on operationalising rights-based contraceptive counselling in low-resource health systems and informs ongoing policy and programme reforms in Pakistan.

The novelty of this paper lies in four linked contributions: operationalising a WHO rights-based counselling lens in routine Pakistani primary care settings; triangulating client and provider perspectives with non-participant observations; including both female and male clients to examine gendered communication and decision-making; and embedding the analysis within a pre-specified multiphase intervention pathway leading to training, job aids, supervision, follow-up and later experimental evaluation.

## Methodology

### Study design

**Figure 1:**
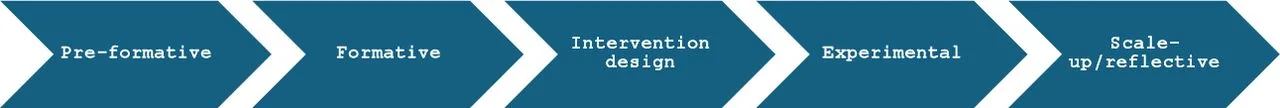
Phases of intervention.

This study forms part of a five-phase complex intervention research programme designed to strengthen contraceptive counselling services in Pakistan. The overall project follows international guidance for the development and evaluation of complex interventions, progressing through five phases as shown below in figure 1:

The present paper reports exclusively on findings from Phase 2 (formative phase), which employed participatory qualitative research methods to explore how contraceptive counselling is currently conceptualised and delivered in routine practice, and to identify context-specific barriers and enablers to rights-based, client-centred counselling.

The formative phase was informed by three complementary frameworks:

- A Theory of Change framework - guided the identification of barriers, enabling factors and pathwaysthrough which the subsequent intervention was expected to improve contraceptive counselling.
- The WHO health-system building blocks/framework - provided a systems-level lens for examining the organisational and service-delivery influences.
- A human rights-based approach to FP articulated by WHO - informed assessment of autonomy, informed choice, privacy, non-discrimination and client-centred counselling.

### Study setting

The study was conducted in Islamabad and Rawalpindi, two metropolitan districts representing diverse urban and periurban primary healthcare contexts in Pakistan. Data were collected from selected public-sector primary care facilities, including rural health centre (RHC) Bharakahu, Islamabad, and RHC Khayaban-e-Sir Syed, Rawalpindi, as well as from a private-sector FP facility operated by the Family Planning Association of Pakistan (FPAP).

These sites were selected during the *preformative phase* through a structured facility mapping exercise using predefined eligibility criteria, including:

- Provided routine FP services.
- Had at least three modern contraceptive methods consistently available.
- Reported no contraceptive stock-outs in the preceding 3 months.
- Recorded a minimum client volume of at least 100 FP visits per month.
- Were not receiving concurrent intensive external technical support for FP.

### Study population and sampling

#### Sampling of clients for in-depth interviews

A facility-based sampling frame was developed at each participating health facility, with support from facility staff. Lists of FP clients who had used any contraceptive method during the preceding 12 months were extracted from facility records. The sampling frame was restricted to recent users because the formative phase aimed to understand actual counselling encounters, method information, side-effect communication, follow-up and continuation-related experiences among those who had recently interacted with services. We recognise that this approach excludes non-users and those who never reached services; this is acknowledged as a limitation. Client lists were stratified by broad contraceptive method category (short-acting methods such as condoms, pills and injectables; long-acting reversible methods such as implants or IUCDs (Intra-Uterine Contraceptive Devices) where available; and permanent/other methods where recorded) to ensure variation in method-related counselling experiences.

Within each method stratum, clients were randomly selected to reduce gatekeeper selection and avoid relying only on easily available or highly satisfied clients. This approach was used because the study sought variation across actual method-use experiences within a defined service-user population rather than theoretical sampling of all possible contraceptive attitudes. Diversity was further supported by balancing women and men across facilities and by including clients using different method categories, age groups and facility types. If a selected participant declined or was unreachable after repeated contact attempts, the next eligible respondent from the same method stratum was approached to maintain method-mix representation.

Recruitment and bias mitigation: Facility staff made initial contact only to introduce the study and obtain permission for research team follow-up. All recruitment conversations and interviews were conducted off-site (neutral community location or private room not accessible to providers) by trained research assistants not affiliated with the facilities. Providers were not informed which of their clients participated. Non-response was tracked: 14% of contacted clients declined (9% in private, 18% in public facilities), with no significant difference by method type. To reduce social desirability, interviewers emphasised that services would not be affected by responses, used neutral phrasing and ensured no facility staff were present during interviews.

Following selection, facility staff made only the initial contact or introduction and did not conduct study interviews. Participation was voluntary, and the research team explained the study objectives, procedures, risks and benefits directly to potential participants before obtaining written informed consent. To reduce social desirability and protect privacy, interviews were conducted by trained research staff rather than service providers, in a private or neutral space chosen by the participant where feasible. Facility providers were not informed about participants’ responses, and identifiable data were not shared with facility staff. Participants were assured that refusal or critical comments would not affect their access to services.

A total of *72 married clients* (36 women and 36 men) were successfully recruited for IDIs across the three facilities:

- FPAP, Islamabad (Private): 12 females and 12 males (n=24).
- RHC Khayaban-e-Sir Syed, Rawalpindi (Public): 12 females and 12 males (n=24).
- RHC Bharakahu, Islamabad (Public): 12 females and 12 males (n=24).

Across the full sample, approximately 60% of participants were aged 19–39 years, and 40% were aged ≥40 years, ensuring representation across reproductive age groups. The client sample was evenly distributed across the three study facilities, with 24 participants recruited from each facility. Each facility contributed 12 women and 12 men, resulting in an overall sample of 36 women and 36 men. In terms of age, 43 participants (59.7%) were aged 19–39 years, while 29 participants (40.3%) were aged 40 years or older. Thus, the sample included a balanced representation by gender and facility type, as well as participation from both younger and older reproductive-age groups. The structured selection process reduced the risk of facility-level gatekeeper selection and supported diversity across gender, age group and public–private facility settings. This structured selection process reduced the risk of facility-level gatekeeper selection and supported diversity across gender, age group, contraceptive-method experience and public–private facility settings.

#### Sampling for observation of contraceptive counselling sessions

For the non-participant observation component, a consecutive sampling approach was employed, in which all FP clients who presented for services during four consecutive days of data collection at each selected facility were eligible for observation, subject to verbal consent. In total, 72 counselling sessions were observed across the three facilities. Observations were conducted in real time, without interfering with provider–client interactions, using a structured checklist adapted from the WHO Service Availability and Readiness Assessment FP module and the Method Information Index (MII). Checklist domains included: (1) method mix discussed; (2) side-effect disclosure (anticipatory vs reactive); (3) client questions invited and addressed; (4) privacy measures; (5) respectful communication; (6) follow-up advice. Observations were conducted in real time without interference in provider–client interactions.^19 20^

#### Sampling of providers and key stakeholders

Healthcare providers, programme managers and policy stakeholders were initially selected using a criterion-based purposive sampling strategy for the wider multiphase study. A total of 35 key informants were recruited across four stakeholder categories: private-sector service delivery organisations (n=7), public-sector officials (n=10), frontline health service providers (n=15) and development partners and donor agencies (n=3). However, the present article analyses only data from frontline service providers, clients and observed counselling sessions. Data from programme managers, public-sector officials, non-governmental organisations and development partners are reserved for a separate analysis of policy, governance and implementation perspectives. This narrower analytical focus strengthens the present paper by concentrating on routine counselling practice as experienced by clients and delivered by providers.

### Data collection and management

Data collection was undertaken from 15 August to 30 November 2024. IDIs were used because contraceptive decision-making, side effects, privacy, coercion and couple communication are sensitive topics. Each IDI lasted a mean of 45 min (range: 20–90 min). Interviews were conducted with an official note-taker present, and monitoring support where required. Written informed consent was obtained before each interview, including specific consent for audio-recording. Where participants declined audio-recording, interviews proceeded with detailed contemporaneous notes and/or written responses. The number of non-audio-recorded interviews was seven. To reduce loss of nuance, note-takers expanded notes immediately after interviews, interviewers debriefed after data collection, and notes were compared with available audio files and field summaries. Female respondents were interviewed by female interviewers where preferred. Interview guides were pilot-tested for contextual relevance and appropriateness before main data collection.

All primary qualitative materials, including verbatim transcripts prepared in the local language, structured field notes from non-audio-recorded interviews and audio files where available were securely transferred to the transcription team for transcription and English translation. The data collection team conducted systematic quality checks of all transcripts and translations for completeness, accuracy and consistency with field notes, resolving discrepancies through reviewer consensus and, where needed, by replaying audio recordings. Finalised transcripts were then imported into NVivo for data management and analysis. The lead technical consultant and second lead data collector established an analytical matrix, organised transcripts by respondent type and thematic domains, and applied the agreed coding structure. Data entry, coding and matrix development were completed in early December 2024.

### Data analysis

Qualitative data were analysed using thematic analysis supported by NVivo software and a hybrid inductive–deductive coding strategy. Two coders (SKA and MAA) independently coded a 20% transcript subset. A codebook was developed inductively from initial open coding, then deductively organised using WHO rights domains (privacy, informed choice, non-discrimination, accountability) and Theory of Change constructs. Disagreements were resolved through consensus with a third reviewer (MA). Coding was followed by axial coding, constant comparison across clients, providers, facility type and gender, and thematic synthesis. Saturation was assessed pragmatically when no substantially new themes emerged within the client and provider groups across the three facilities. Themes were then mapped to rights domains, including informed choice, privacy and confidentiality, non-discrimination, respectful care, accountability and follow-up. Negative or deviant cases, such as clients reporting adequate public-sector counselling or providers describing constraints despite rights-based intentions, were examined to avoid overgeneralisation.

Researcher reflexivity and positionality: The research team included public health specialists, social scientists and clinicians. No team member had prior working relationships with the participating public facilities; the private FPAP facility was known professionally, but the research team maintained independence by using external data collectors and emphasising confidentiality. Female interviewers interviewed female clients, and interviews were conducted in community settings to minimise hierarchical dynamics. An audit trail (NVivo project archived, coding matrices available from corresponding author) was maintained.

### Patient and public involvement

Patients and members of the public were not involved in the design, conduct, reporting, or dissemination plans of this research.

## Results

The formative phase was conducted at three primary-level FP facilities in Islamabad and Rawalpindi: two public-sector RHCs and one private facility. The analysis presented here includes 72 client IDIs, 15 frontline provider IDIs and 72 non-participant observations of counselling encounters. Data from policy stakeholders and development partners were collected for the broader multiphase project but are intentionally excluded from this paper to maintain focus on routine counselling practice at the point of care.

Across respondent groups, there was broad agreement that ‘counselling’ is routinely reported as part of FP services; however, closer examination revealed that counselling was often equated with the provision of basic information on methods rather than a structured, client-centred process as defined in the study protocol and in Ali *et al*.^8 13 14^

Eight pre-specified areas of inquiry were explored through the interview guides and observation checklist. These pre-specifed areas became topic for data collection, not the final analytical themes. They included participants’ overall counselling experiences, collaboration and exchange of information, discussion and understanding of side effects, follow-up expectations, suggestions for improving services, access to easy-to-understand evidence-based information, coercion-free informed choice and privacy during counselling encounters. The analytical themes presented below were generated through thematic synthesis across interviews and observations.

### Theme 1: collaboration, information exchange and client experience of contraceptive counselling

#### Subtheme 1.1: client experience of counselling – public–private contrasts

Clients’ narratives clearly demonstrate *a strong public–private contrast in the content and depth of counselling*. Private-sector clients frequently described exposure to *multiple methods, visual tools and couples counselling*: *“*We were shown videos during the counselling session, which made it easier to understand the different methods. This was something I didn’t experience at the public facility.*” (Female client, private-sector-ISB*). Another female respondent from private sector, in relation to basket of contraceptive method revealed: *“*The health facility provided me with a wide range of family planning options—pills, injections, implants, condoms, even surgery. It gave us the flexibility to choose what was best for us.*” (Female client, private-sector-ISB), the same respondent further added about the couple counselling and shared, “*Couple counselling was really helpful. Both of us could ask questions and get clear answers, which made the decision-making process much smoother.*” (Female client, private-sector-ISB*).

In contrast, public-sector clients often reported receiving limited and method-specific information, primarily focused on condoms, tablets and injections: *“*We were informed about some family planning methods like condoms and tablets.*” (Female client, public-sector-ISB). In the same context another respondent added, “*First, she explained the use of condoms, and then there were tablets and injections—just a few easy options.*” (Female client, public-sector-ISB*).

Misclassification of ‘information giving’ as ‘counselling’ was common: *“*Yes, I received family planning counselling, but I wasn’t aware of it before, the service provider informed me about condoms.*” (Male client, public-sector-RWP*).

Clients also described *provider-led method steering based on side-effect fears or availability constraints. We distinguish three forms: (1) perceived steering (client accounts of providers directing choice*): *“*The doctor said the side effects were causing problems for my family, so she suggested using condoms to avoid the complications caused by other methods like tablets or rings, which can lead to pain or side effects.*” (Female client, public-sector-RWP*); (2) structural steering (provider accounts of stock-outs): “We try not to pressure clients, but there are times when certain methods are not available, so we push the ones we have. That’s not ideal.” *(Service provider, public-sector-ISB)*; (3) intentional coercion (no direct evidence, though subtle pressure related to targets was reported).

Across both sectors, clients and providers equated ‘counselling’ primarily with method-focused information sharing rather than with a structured, preference-sensitive, rights-based decision-making process. While private facilities offered relatively broader method choice, audiovisual tools and couple-centred engagement, public facilities remained constrained by high patient loads, insufficient educational materials and commodity limitations. The data further indicate subtle method steering linked to side-effect anxieties and stock availability, which compromises the ideal of fully informed, autonomy-driven contraceptive choice.

Observation data corroborated this public–private contrast. In observed public-sector encounters, counselling was often brief and focused on a limited number of available methods, while private-sector observations more often documented visual aids, more time for questions and broader discussion of options. However, observation also showed variation within sectors, indicating that facility resources, provider workload and consultation space shaped counselling quality alongside ownership type.

To avoid overinterpreting intent, we use the term ‘steering’ in three distinct ways: perceived steering when clients felt guided toward a method; structural steering when limited method availability or workload narrowed the options discussed; and intentional coercion only when evidence suggests pressure or disregard for voluntary choice. Most examples in this study reflected perceived or structural steering rather than explicit coercion.

#### Subtheme 1.2: collaborative information exchange and client engagement

Service providers across both public and private sectors consistently framed counselling as a collaborative process, emphasising the importance of explaining available methods, assessing prior knowledge and encouraging client questions. A private-sector provider described this approach as follows: *“*Yes, I believe we give them the information they expect. We try to explain everything in detail, especially the methods that are available. We also ask if they have any prior knowledge or preferences to guide our counselling.” *(Service Provider, private-sector-ISB*). Similarly, a public-sector provider highlighted the need to build on clients’ existing expectations *“*We do our best to explain the different methods and answer all their questions. Sometimes they come with expectations already, so we build on that.” *(Service Provider, public-sector-ISB*).

Providers repeatedly stressed the importance of *creating a safe, open environment for dialogue*, particularly around sensitive issues such as side effects and cultural concerns: *“*Yes, we always encourage them to ask questions. It’s important for them to feel comfortable, and sometimes they have concerns about side effects or cultural issues. We let them know it’s okay to ask anything.” *(Service Provider, private-sector-ISB*).

However, this ideal of collaborative engagement was structurally constrained in the public sector due to high patient volumes and limited consultation time: *“*In most cases, yes. We ask them if they have any concerns or questions before we proceed. But with many patients in the clinic, sometimes there’s not enough time for a long discussion.” *(Service Provider, public-sector-RWP*).

Beyond method-level information, providers also described positioning FP within a broader well-being and maternal–child health narrative: *“*We focus on how family planning improves the health of the mother and children. We let them know it’s not just about contraception, it’s about family well-being and making sure every child is cared for.” *(Service Provider, private-sector-ISB). “*We explain how family planning helps prevent unwanted pregnancies and benefits women’s health. It gives women time to recover between pregnancies, which is crucial for their long-term health.” *(Service Provider, public-sector-RWP*).

Analytically, these accounts reflect strong provider commitment to information sharing and client engagement, but also reveal clear health-system constraints, particularly time pressure in public facilities, that limit the depth of collaboration and sustained dialogue.

### Theme 2: communication of side effects and informed choice

Across clients and providers, side effects emerged as a central yet inconsistently addressed component of contraceptive counselling, with substantial variation in how risks were communicated and understood. While most participants recalled that side effects were mentioned during counselling encounters, the depth, clarity and balance of these explanations differed markedly across facilities and between genders. Several clients confirmed that side effects were acknowledged, but often in a limited or non-detailed manner: *“*Yes, they explained that side effects may occur.” *(Female client, public-sector-ISB*). To be hesitant of shyness emerged as a barrier which also restricted to inquire about the side effects as well. As it was shared one of the male respondents from Rawalpindi, *“*Yes, side effects were mentioned, while accompanying with my wife, but I didn’t ask for more details.” *(Male client, public-sector-RWP*).

In some cases, information was conveyed in a highly partial or reactive way, focusing only on what to do if problems emerged rather than proactively discussing risks: *“*No, no, she mentioned that if I experienced bleeding or a stomach-ache, I should take these pills.” *(Female client, public-sector-RWP*).

This selective communication pattern sometimes resulted in method switching driven by fear of complications, rather than by structured risk-benefit discussions: *“*First service provider suggested IUCD for me, however, when I shared my health concerned in detail then she suggested other method” *(Female client, publc-sector-ISB). “*Yes, but I preferred the tube method, so I chose it even though they told me it has more side effects than benefits for my body.” *(Female client, public-sector-ISB*).

Providers across both public and private sectors described adopting a *‘balanced’ communication strategy*, in which side effects were disclosed but deliberately framed to avoid alarming clients: *“*We are upfront about side effects. I tell them every method has some side effects, but they’re usually not serious and can be managed. It’s important for them to know what to expect, but I reassure them it’s nothing to worry about in most cases.” *(Service Provider, private-sector-ISB*).

“We explain the side effects in a way that doesn’t scare them. Some clients get nervous when they hear about side effects, so we try to keep it balanced and let them know that they can switch methods if something doesn’t work for them.” *(Service Provider, public-sector-ISB).*

While this approach reflects provider concern for client anxiety and continuation, it also illustrates how risk communication is shaped by fears of discouraging uptake, rather than being consistently guided by informed-consent standards.

Several client narratives indicate that risk communication was sometimes framed selectively to steer method choice, particularly in contexts of prior health concerns or perceived family sensitivities: *“*The doctor said the side effects were causing problems for my family, so he suggested using condoms to avoid the complications caused by other methods like tablets or rings, which can lead to pain or side effects.” *(Female client, public-sector-RWP).*

This pattern highlights how side-effect narratives can become a mechanism for method steering, rather than a neutral foundation for autonomous decision-making. Taken together, these findings show that side effects are widely acknowledged but unevenly operationalised within counselling encounters. Women’s greater proactivity in raising side-effect concerns reflects their disproportionate exposure to bodily risks, while men’s more passive engagement suggests important gendered gaps in contraceptive responsibility and risk perception. At the provider level, the tension between full transparency and fear of deterring uptake shapes a form of *partial disclosure*, which may unintentionally undermine informed consent and long-term method continuation. The data further suggest that method switching is sometimes driven by anxiety-based counselling rather than structured, evidence-based risk communication.

### Theme 3: collaboration, information exchange and barriers to meaningful dialogue

Providers across both sectors also emphasised that they routinely encourage open dialogue, assess clients’ prior knowledge and attempt to tailor counselling accordingly. One provider explained, *“*We do our best to explain the different methods and answer all their questions. Sometimes they come with expectations already, so we build on that” *(Service Provider, public-sector-ISB)*. Another added, *“*We always encourage them to ask questions… especially if they have concerns about side effects or cultural issues” *(Service Provider, private-sector-ISB)*. These accounts reflect a shared normative commitment to client-centred counselling and two-way communication.

However, these ideals were unevenly realised in practice, particularly in the public sector. High patient volumes and limited staffing created structural constraints that curtailed meaningful dialogue. As one public-sector provider acknowledged, *“*Time is a big barrier, especially in busy clinics. We try our best, but there’s only so much we can cover when many patients are waiting” *(Service Provider, public-sector-ISB)*. Even when providers attempted to invite questions, clinical pressures often resulted in *compressed, one-way information delivery* rather than genuine shared decision-making.

Gendered communication norms further shaped information exchange. Several men described feeling reluctant to speak during consultations, even when present with their wives. One male client explained, *“*I visited the health facility with my wife. Doctor was polite and explained us about the methods. But I could not ask questions, I was shy and felt hesitation” *(Male client, public-sector-RWP)*. Providers echoed this dynamic, particularly in rural settings: *“*Sometimes the clients themselves are hesitant to ask questions. In rural areas, women are shy to talk about family planning, and men often don’t come to counselling sessions” *(Service Provider, private-sector-ISB)*. These patterns limited the depth of spousal engagement and collaborative decision-making, despite formal encouragement of participation.

Private-sector providers were more likely to describe adaptive strategies to overcome time and communication barriers, including the potential use of digital tools and stronger partner involvement. As one provider noted, *“*We need more time with each client, and maybe more use of digital tools to follow up. Also, involving the husband in counselling sessions could improve decision-making” *(Service Provider, private-sector-ISB)*. Such strategies were rarely feasible in high-volume public facilities.

Overall, while collaborative communication and client autonomy were widely endorsed in principle, their routine implementation was constrained by systemic pressures (time, workload), gender norms (male silence, female modesty) and facility-level resource differences. These tensions shaped whether counselling functioned as a two-way deliberative process or remained a provider-led exchange of basic information, with important implications for informed choice and method continuation.

Observation findings helped distinguish reported practice from enacted practice. Providers often described encouraging client questions and protecting privacy; however, direct observations showed that high client volume, shared consultation spaces, interruptions and limited use of counselling aids frequently constrained the depth and quality of counselling. These findings were consistent with the observation checklist domains on rapport-building, open-ended questioning, confidentiality, client engagement, tailored method discussion, side-effect counselling, confirmation of understanding and follow-up advice. Observations therefore strengthened the interpretation that counselling gaps were not only individual provider issues but were also shaped by service organisation, workload, infrastructure and availability of counselling materials.

Gendered patterns were also evident when observation findings were interpreted alongside client and provider interviews. Women more frequently raised concerns about side effects, bodily symptoms, privacy and continuity of care, while men often described themselves as supporters of FP decisions but reported embarrassment or limited participation in detailed method discussions. Observed encounters further showed that couple counselling was valued where available because it allowed both partners to ask questions and clarify concerns. However, its absence in many public-sector encounters meant that gendered decision-making dynamics were not routinely addressed during counselling.

### Theme 4: follow-up and continuity of care

Few facilities demonstrated structured and proactive follow-up systems. This was most evident in the private sector, where some providers described issuing follow-up cards and scheduling return visits as part of routine care. As one private provider explained, *“*We give them a follow-up card and schedule their next visit. It’s important to see how they’re doing, especially if they’ve just started using a new method” *(Service Provider, private-sector-ISB)*. In contrast, public-sector follow-up relied largely on verbal advice, with the burden placed on clients to re-initiate contact if problems arose. A public provider noted, *“*We tell them to come back in case of any issues, or after a certain period to check how things are going. But usually they don’t return, especially in rural areas” *(Service Provider, public-sector-RWP).*

Most clients confirmed that they were verbally advised to return if they experienced complications, but *systematic* follow-up mechanisms were weak, especially in public facilities. Several clients recalled being told they could come back if needed: *“*Yes, they told us we could return if needed. If there’s any problem, we can contact them” *(Female client, public-sector-ISB)*, and *“*If there’s any complication, the service provider told me I could return” *(Male client, public-sector-RWP)*. However, very few reported receiving written reminders or formal documentation, reinforcing the ad hoc nature of follow-up in routine practice.

Clients who returned for follow-up, most often due to side effects, generally expressed relief and satisfaction with the care they received. One woman reflected, *“*They told me to come back if I had any issues. I’ve been well taken care of and would return” *(Female client, public-sector-ISB)*. Another male client described how follow-up enabled method discontinuation and symptom management: *“*After five or six months, my wife experienced stomach pain and pain while urinating. We consulted the doctor, and they advised us to stop using the condom. Now we mostly use condoms because they are cheaper and cause fewer infections” *(Male client, public-sector-ISB)*. These accounts highlight that when follow-up did occur, it played a critical role in managing side-effects, modifying method use and rebuilding client confidence.

Providers described more organised follow-up practices in some private organisations, including reminder cards and informal tracking systems, compared with the fragmented and resource-constrained approaches in the public sector, where high patient volumes and underused digital systems limited continuity of care.

### Theme 5: privacy, confidentiality, respect and dignity

Most clients reported that privacy was generally respected during FP consultations, particularly in private-sector facilities, where one-to-one encounters and enclosed rooms were more common. Several clients explicitly described private, uninterrupted interactions with providers. One woman noted, *“*Yes, they have taken care of it. We met one to one. There was no further patient. They took care of privacy a lot” *(Female client, private-sector-ISB)*. Similarly, a male respondent stated, *“*They have respected us and gave us the documents and files. They gave me respect… He told me everything. I liked it. He gave me respect” *(Male client, private-sector-ISB)*. These accounts indicate that privacy and respectful communication were closely linked in clients’ perceptions of quality care.

However, privacy breaches were frequently reported in public-sector facilities, largely due to overcrowding, shared spaces and fragile infrastructure. Counselling often occurred in semi-open areas or corners near outpatient departments, with thin partitions or nearby patients. One respondent articulated the strong social sensitivity attached to confidentiality: *“*Families who are sitting there should not be discussed in front of them. Individually, they should discuss things with the family… Neither should they share things with any other family” *(Female client, public-sector-ISB)*. This highlights that privacy was not only a technical requirement but also a deeply moral and social expectation, tied to honour, family reputation and gender norms.

Religious and cultural sensitivities further shaped how privacy was experienced. One male participant reflected, *“*People who are more religious… they don’t like this. You know, it varies from person to person” *(Male client, public-sector-RWP)*, suggesting that the threshold for acceptable disclosure during counselling varied considerably across clients, reinforcing the importance of adaptable, context-sensitive privacy arrangements.

Stakeholders across sectors recognised confidentiality as a core ethical principle, yet openly acknowledged that it was routinely compromised in practice. Key informants pointed to *open* discussions in front of accompanying relatives, visible client records and corridor-based service provision as structural contributors to privacy erosion. One provider explicitly described the infrastructural limitations: counselling in ‘separate cabins’ was frequently identified as the ideal but unrealised standard, with one female participant emphasising, *“*The best way to improve privacy is to provide separate cabins where each person can talk privately” (Female client, public-sector*-ISB).*

*Providers described respect and dignity as aspirational profess*ional values, yet their routine enactment remained uneven. Providers consistently asserted their commitment to respectful care. As one stated, *“*I always try to treat my clients with the utmost respect. I listen to their concerns attentively and address them without judgment” *(Service Provider, public-sector-ISB)*. Another added, *“*By considering their personal circumstances and cultural backgrounds, we show that we value their autonomy and personal choices” *(Service Provider, public-sector-RWP).*

### Theme 6: training, tools and system-level barriers

Across public, private and development partners, there was consensus that current provider training and supervision are insufficient to support high-quality, rights-based counselling. While Pakistan’s FP curriculum references GATHER, BCS and C4C, these are not systematically implemented, and refresher trainings rarely address real-world constraints. Frontline staff reported struggling to translate idealised training scenarios into high-volume facility environments.

Time constraints, human resource shortages and supply-chain problems were consistently cited as structural barriers. Stock-outs led providers to steer clients towards whatever methods were available, undermining the principle of method choice.

Language and literacy barriers, especially in rural areas, further impeded effective counselling; providers frequently resorted to medical jargon that clients could not understand. Stakeholders recommended wider use of visual aids and locally adapted materials, as well as structured feedback mechanisms to track whether counselling content is actually understood.

Finally, pressure to meet numerical FP targets was seen as a driver of subtle coercion: providers sometimes felt compelled to prioritise method uptake over client preferences, which could translate into biased counselling and compromised informed choice.

## Discussion

This formative multimethod qualitative study highlights substantial gaps between the policy ideal of rights-based, client-centred contraceptive counselling and the realities of routine service delivery in three primary care facilities in Islamabad and Rawalpindi. Some findings, such as weak follow-up systems, side-effect fears and target-driven programme pressures, are consistent with wider evidence from Pakistan. Other findings, particularly the degree of public–private contrast and facility-specific privacy constraints, should be interpreted as context-specific to the study sites and transferable mainly to comparable urban and periurban primary care settings.^21^

Local and global evidence consistently links high-quality counselling with improved informed choice, satisfaction and contraceptive continuation.^22 23^ Our findings reinforce the concern that deficiencies in counselling content and process remain a key barrier to closing Pakistan’s persistent gaps in contraceptive uptake and sustained use.

Client narratives revealed a clear public–private contrast in the depth and breadth of counselling. Private-sector facilities were more likely to offer a wide method mix (including implants and permanent methods), use audiovisual tools and support couples’ decision-making, while public facilities tended to focus on a narrow range of short-acting methods, often delivered as one-way information. These findings align with recent work demonstrating that Pakistan’s private and social franchise sectors provide a substantial share of FP services, often with expanded method availability and more client-oriented delivery models.^21^

However, across both sectors, ‘counselling’ was widely misclassified as any FP-related information, even when core elements of rights-based counselling, eliciting preferences, explaining trade-offs and supporting autonomous choice, were absent. This echoes evidence from global reviews and rights-based FP assessments showing that informed choice is frequently undermined by partial information and directive communication, despite formal endorsement of rights-based approaches.^3 24^ The findings of this formative research study therefore reinforce WHO guidance that rights-based FP cannot be reduced to method description alone but requires a structured, preference-sensitive process that respects autonomy, non-discrimination and non-coercion.^19 25^

Providers across both sectors described counselling as collaborative, emphasising efforts to build on clients’ prior knowledge and encourage questions. In practice, however, this ideal was often constrained by high patient volumes and limited consultation time, particularly in public facilities, resulting in compressed, provider-led encounters rather than genuine shared decision-making. Similar tensions between normative commitment and operational feasibility have been reported in evaluations of counselling quality using the MII.^22 26^

Gendered dynamics of communication were prominent. Women were more likely to actively raise concerns about side effects, while men often reported feeling shy or reluctant to speak during sessions. Emerging evidence from Pakistan shows that male involvement in FP remains limited and shaped by restrictive gender norms, despite its recognised importance for shared decision-making.^27–29^ Interventions that seek to promote male engagement must therefore carefully balance couple participation with the protection of women’s autonomy and decision-making power.^30^

Side effects emerged as a central yet inconsistently addressed component of counselling. While most clients recalled that side effects were mentioned, disclosure was often partial and reactive rather than anticipatory. Providers described deliberately ‘balancing’ information to avoid frightening clients and discouraging uptake. This selective disclosure is problematic, as poor side-effect counselling is a well-established driver of early discontinuation.^22 31^

Our findings also resonate with newer qualitative evidence on contraceptive self-care and autonomy, which emphasises transparent, evidence-based side-effect counselling as foundational for informed choice.^25^ Moreover, side-effect narratives were sometimes used to steer method choice, particularly by framing long-acting methods as ‘risky’ and promoting condoms as a compromise, raising concerns under a human-rights framework where non-coercion and voluntary choice are central obligations.^3 24^

Follow-up systems were generally weak and ad hoc in public facilities, where clients were verbally advised to return if problems occurred but rarely received scheduled appointments or reminders. In contrast, some private facilities used structured follow-up tools such as cards and reminder visits. Evidence on quality-adjusted FP coverage highlights continuity, side-effect management and follow-up as integral components of service quality, rather than optional add-ons.^22 23^

In Pakistan, recent political economy and bottleneck analyses highlight fragmented follow-up systems, weak data use and limited digital integration as constraints on programme effectiveness.^31 32^ Our findings support the urgent need to institutionalise simple, feasible follow-up mechanisms within public-sector FP services.

While privacy and respect were more consistently reported in private facilities, breaches were common in overcrowded public hospitals where counselling occurred in shared or semi-open spaces. Clients linked privacy not only to comfort but also to social reputation, honour and religious norms, indicating that breaches may carry significant social consequences. These findings align closely with global quality and rights frameworks that identify privacy, confidentiality, dignity and non-discrimination as non-negotiable elements of FP services.^3 19 33^

Pakistan-specific assessments similarly call for infrastructure-level investments such as dedicated counselling rooms and redesigned client flow, to ensure confidentiality and respectful care.^31^

Interpreted through WHO rights-based counselling domains, the findings point to several actionable gaps. Informed choice was weakened when counselling was equated with basic information or when side effects and alternatives were discussed unevenly. Privacy and confidentiality were compromised by shared spaces and interruptions, especially in public facilities. Non-discrimination and respectful care require attention to gender, literacy and socio-economic differences in how clients are addressed and supported. Accountability requires routine monitoring of client experience, coercion-free choice, follow-up and method switching rather than only counting method uptake.

### Implications for the intervention package

**Figure 2:**
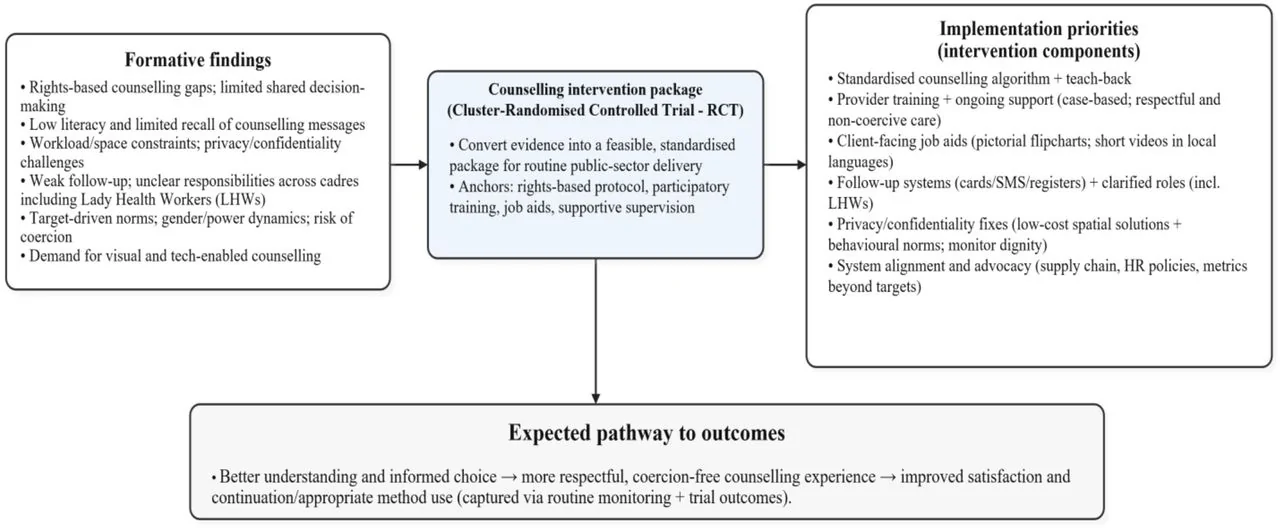
Conceptual diagram of the intervention package.

The formative findings directly inform the design of the contraceptive counselling intervention package to be tested in the subsequent cluster randomised trial. The specific components shaped by this formative work are: (1) a pictorial job aid covering method comparison, side-effect management and teach-back; (2) a 1-day provider training module (case-based, rights-focused, including gender and power dynamics); (3) a follow-up card and SMS reminder protocol; (4) a supervision checklist with rights-based indicators. Below is the conceptual diagram (refer to figure 2) of the proposed intervention package:

Several priorities emerge:

#### Standardised, rights-based counselling protocol

- Develop a concise, context-appropriate counselling algorithm that operationalises the study’s rights-based definition, including steps to elicit client needs and preferences, present the full method mix (within supply constraints), discuss side-effects transparently and support shared decision-making.
- Integrate simple ‘teach-back’ or feedback techniques to confirm client understanding, particularly in low-literacy settings.

#### Practical training and ongoing support for providers

- Move beyond one-off didactic trainings towards participatory, case-based sessions that reflect real workload and space constraints in public facilities.
- Include modules on gender and power, respectful care and non-coercive counselling under target-driven programmes, alongside technical updates.
- Establish feedback mechanisms (eg, supervisory checklists, peer reflection meetings) to close the loop between training and practice.

#### Tools to support understanding and recall

- Co-design visual aids, pictorial flipcharts and short videos in local languages that can be used consistently across sectors, responding to client suggestions for more visual and technology-enabled counselling.

#### Strengthened follow-up systems

- Adapt proven private-sector practices (eg, follow-up cards, SMS reminders, simple registers) to public facilities, with attention to feasibility and data burden.
- Clarify responsibilities for follow-up across facility-based and community-based providers, including female health workers.

#### Structural attention to privacy, confidentiality and respect

- Incorporate low-cost spatial solutions (curtains, screens, designated corners) and clear behavioural norms for respecting confidentiality even when dedicated rooms are not available.
- Embed indicators on respect, dignity and coercion-free choice into routine monitoring and the trial’s outcome framework.

### System-level advocacy and alignment

- Use these findings in dialogue with provincial and national stakeholders to align supply-chain management, human-resource policies and performance metrics with the goal of client-centred, rights-based counselling rather than numeric targets alone.

### Thematic summary of formative findings on contraceptive counselling

These findings (see table 1) align with the global literature emphasising the importance of tailored, high-quality counselling to improve method continuation and user satisfaction.^1 4^

**Table 1:** Thematic summary of formative findings on contraceptive counselling

Respondent group	Clients’ experience with counselling	Content and depth of contraceptive counselling	Collaboration, information exchange and client autonomy	Side-effects communication and informed choice	Follow-up and continuity of care	Privacy, confidentiality, respect and dignity	Training, tools and system-level barriers
**Clients—key findings**	Clients from public and private facilities generally reported receiving ‘counselling’, but this was usually understood as any FP-related discussion rather than a structured, client-centred process; experiences varied by facility type and provider workload.	Most women and men reported brief information on a limited range of methods (mainly condoms, pills, injectables). Private facilities were perceived to offer more comprehensive information, including long-acting methods and permanent options. Couple counselling was more common in private settings and highly valued by clients.	Many clients described positive, collaborative interactions, particularly in better-resourced settings where they felt able to ask questions. Some men, however, reported feeling shy and limited their participation in discussions due to gender norms. Clients valued being ‘given freedom to choose’ after explanation of pros and cons.	Clients often recalled that side-effects were mentioned but with variable depth. Women were more proactive than men in raising questions about side-effects. Fear of side-effects, unmanaged problems and lack of clear explanations contributed to anxiety and, in some cases, discontinuation.	Most clients were told they could return if they faced problems, but formal follow-up (eg, scheduled appointments, reminder cards) was rare, especially in public facilities. Those who did return and receive support for side-effects or method switching were generally satisfied.	Privacy was better in private facilities, where consultations often occurred in separate rooms. In many public facilities, counselling took place in semi-open spaces, limiting auditory and visual privacy. Despite this, clients commonly perceived providers as respectful, though some noted rushed or brusque interactions in crowded settings.	Clients reported that providers sometimes used technical language that was difficult to understand. Limited or confusing information, combined with stock-outs, reduced the sense of genuine choice. Some clients perceived that better-off or more educated clients were treated more attentively.
**Providers/system—key findings**	Providers and key informants widely asserted that counselling is part of routine FP services. Still, they recognised that day-to-day practice rarely aligns with ideal rights-based definitions due to time, space and workload constraints.	Providers, particularly in private facilities, believed they provided ‘complete’ counselling but acknowledged that time pressure often leads to truncated discussions. Public providers frequently steer clients towards a limited set of methods, sometimes influenced by availability and performance targets.	Policymakers and development partners confirmed that genuine shared decision-making is not consistently achieved, particularly in high-volume public facilities. Some stakeholders noted continued use of directive or fear-based messaging in certain settings, despite global guidance to avoid such approaches.	Providers expressed ambivalence about disclosing detailed side-effect information, fearing this might discourage uptake. Donors and academics, however, stressed that inadequate disclosure undermines informed choice and contributes to dissatisfaction and discontinuation. Use of evidence-based figures or simple risk communication tools was reported but not widespread.	In public facilities, follow-up systems were largely ad hoc; staff added follow-up advice verbally without systematic tracking. Some private organisations had more structured systems (eg, reminder cards, phone calls). Development partners highlighted follow-up as essential for both method continuation and programme data quality.	Stakeholders agreed that confidentiality is a stated value but often compromised in practice because of cramped infrastructure, lack of separate rooms and heavy client flow. Differential treatment of clients by socio-economic status was reported as a persistent concern.	Training curricula reference tools such as GATHER and BCS but these are inconsistently implemented. Refresher trainings rarely address real-world workload, supply-chain problems and target pressure. Stock-outs, human resource shortages, language and literacy barriers, and numeric FP targets were all seen as major structural barriers to rights-based, client-centred counselling.
**Implications for intervention design**	Clarify and socialise a shared, rights-based definition of contraceptive counselling among clients, providers and policymakers. Design the intervention to move from generic information-giving towards a structured, client-centred process.	Introduce a concise counselling protocol and job aids that support providers to cover the full method mix and key content within time constraints. Promote couple counselling where feasible and ensure public-sector providers can offer a wider range of options when supplies permit.	Embed techniques for eliciting client preferences, encouraging questions and supporting autonomous decision-making. Include gender-focused and communication-focused content in provider training, with explicit strategies to engage men without undermining women’s autonomy.	Standardise transparent discussion of side-effects using simple, evidence-based messages. Emphasise that clients can switch methods and that managing side-effects is part of quality care, not a failure of the method.	Adapt feasible follow-up systems (cards, SMS, simple registers) for public facilities and clarify provider roles for follow-up and method switching. Integrate follow-up indicators into routine monitoring and the trial’s process evaluation.	Incorporate low-cost solutions (curtains, screens, clearer norms) to enhance privacy and confidentiality. Address respectful care explicitly in training and supervision, with indicators on respect and non-coercion in the monitoring framework.	Strengthen practical, hands-on training and ongoing supervision that acknowledge system constraints. Align supply-chain management and performance metrics with rights-based counselling goals to reduce pressure for coercive or target-driven practices.

BCS, Balanced Counselling Strategy; FP, family planning; GATHER, GATHER is a six-component, client-centred FP counselling approach—Greet, Ask, Tell, Help, Explain, and Return/Refer.

### Implications for policy and practice

1. *Operationalising rights-based counselling frameworks:* Frameworks such as GATHER, BCS and human rights-based FP must be translated into practical job aids, supervision tools and performance indicators to support structured, preference-sensitive, non-coercive counselling under time constraints.
2. *Strengthening gender-responsive communication:* Risk communication on side effects must be anticipatory, transparent and evidence-based, while addressing gendered barriers that limit male participation without compromising women’s autonomy.
3. *Embedding follow-up into routine care:* Public-sector services should adopt feasible follow-up mechanisms such as appointment cards, registers and SMS reminders, building on models used in the non-governmental organisation and private sectors.
4. *Addressing structural determinants of quality:* Investments in staffing, commodity security, private counselling spaces and performance metrics that prioritise quality over numeric targets are essential to align FP services with rights-based commitments.
5. *Redesign training curricula:* Incorporate practical, scenario-based training on rights, side effects and non-directive dialogue.
6. *Develop standardised tools:* Visual job aids, method comparison charts and checklists in local languages.
7. *Introducing couple counselling protocols:* Especially in the private sector.
8. *Strengthen supervision and monitoring:* Include qualitative indicators of respectful, informed care.
9. *Ensure follow-up systems:* Via LHWs (Lady Health Workers) or digital tools (eg, SMS, mobile apps).
10. *Address stockouts:* Avoid method-specific bias in counselling due to limited availability.

The forthcoming experimental phase, comparing routine care, a counselling package alone and the same package plus wider method availability, will provide robust evidence on the effectiveness of a multi-component intervention in improving decision-making autonomy, met need and modern contraceptive use. Process evaluation will be critical to understand implementation fidelity, provider and client experiences, and the interplay between counselling quality and structural constraints such as stock-outs and staffing.

At the programme level, these findings point to the importance of embedding counselling strengthening within broader efforts to improve FP quality of care while linking training, supervision, commodity security, community engagement and respectful maternity care initiatives.

### Implications for future research

Future research should evaluate integrated, multicomponent counselling interventions using mixed-methods and implementation science approaches to assess their impact on autonomy, continuation and equity across Pakistan’s diverse health system settings.

### Strengths and limitations

Strengths of this study include its multi-method qualitative design, triangulating client and provider interviews with non-participant observations across public and private facilities. The study included equal numbers of female and male clients, allowing attention to gendered communication and decision-making. It was embedded in a prespecified multiphase intervention pathway, enabling direct translation of formative findings into training, job aids, follow-up and supervision components.

Limitations include restricted transferability due to the focus on three urban/periurban facilities in Islamabad and Rawalpindi; findings may not reflect rural, conflict-affected or highly underserved areas. The facility-record sampling frame included recent contraceptive users and therefore did not capture women, men or couples who needed contraception but never accessed services or discontinued before appearing in facility records. Initial contact through facility staff may have introduced selection or social desirability bias, although the research team conducted interviews with confidentiality safeguards. Some interviews relied on detailed notes rather than audio-recordings, which may have reduced nuance. Observational checklists also may not fully capture subtle communication dynamics or unspoken power relations.

## Conclusion

This formative phase shows that contraceptive counselling is widely recognised and generally valued by clients in the participating facilities in Pakistan, but it often functions as method information rather than as a structured, rights-based process supporting autonomous reproductive decision-making. The findings identify actionable gaps in informed choice, side-effect communication, privacy, follow-up and provider support. They directly inform a context-sensitive counselling package that will combine rights-based protocols, provider training, visual job aids, supportive supervision, privacy safeguards and follow-up systems. Transferability should be considered in relation to similar public and private primary care settings in Pakistan.

## Supplementary Material

Reviewer comments

Author's manuscript

## Data Availability

Data are available upon reasonable request. All data relevant to the study are included in the article or uploaded as supplementary information. Data are available upon reasonable request. Data are available upon reasonable request from the corresponding author.

## Footnotes

Non-participant observations of counselling sessions were also conducted as part of the broader formative phase; however, these data are not analysed in the present paper.
