# Supplementary material for: Closing the counselling gap: a qualitative study to strengthen rights-based family planning in Pakistan
Source: BMJ Open. 2026 Aug 24;16(8):e116059. doi: 10.1136/bmjopen-2025-116059 (PMC13536155; doi:10.1136/bmjopen-2025-116059)
Supplement: Supplementary file 2 [file bmjopen-2025-116059.reviewer_comments.pdf]

# PEER REVIEW HISTORY

BMJ Open publishes all reviews undertaken for accepted manuscripts. Reviewers are asked to complete a checklist review form and are provided with free text boxes to elaborate on their assessment. These free text comments are reproduced below.

## ARTICLE DETAILS

### Title (Provisional)

Closing the Counselling Gap: A qualitative study to strengthen rights-based family planning in Pakistan.

### Authors

Azmat, Syed Khurram; Awan, Muhammad Ali; Thom, Ellen Mpangananji; Uzma, Qudsia; Sheikh, Imran; Kapustianyk, Grace; Kiarie, James; Ali, Moazzam

---

## VERSION 1 - REVIEW

---

| | |
|---|---|
| **Reviewer** | **1** |
| **Name** | **Tikmani, Shiyam** |
| **Affiliation** | **Aga Khan University, Community Health Sciences** |
| **Date** | **15-Jan-2026** |
| **COI** | **None** |

---

bmjopen-2025-116059

Family planning counselling is a core determinant of contraceptive uptake, continuation, and—critically—women's and couples' reproductive autonomy, so evidence that clarifies what "counselling" looks like in routine practice (and why it fails) is highly policy-relevant in Pakistan. This manuscript addresses that gap through a formative, rights-based qualitative assessment across public and private primary care facilities in Islamabad/Rawalpindi, intended to inform a multi-phase intervention package.

Major comments

1) Abstract - inconsistencies

1. The abstract frames the study as "mixed-methods participatory" (IDIs + observations + thematic analysis). In the manuscript, the primary analytic emphasis appears qualitative; if "mixed methods" is intended, clarify what the quantitative element is (if any), how it was analysed, and how integration occurred. Otherwise, rephrase to "multi-method qualitative" (IDIs & observations).

2. The abstract lists clients, providers, and 10 stakeholders; later, the methods state that broader stakeholder KIIs were collected but excluded from this paper's analysis. The abstract

should explicitly state which respondent groups are included in the analysis (clients + providers + observations) and which are excluded.

3. Statements implying national-level insufficiency are plausible but should be framed as findings from three facilities in two districts, not as definitive “Pakistan’s current model” unless clearly supported by broader evidence.

2) Introduction

4. The introduction is conceptually strong (rights-based framing), but it would benefit from a more explicit logic chain: (a) discontinuation/unmet need in Pakistan (b) counselling quality affects autonomy/continuation (c) limited empirical evidence triangulating client/provider perspectives across sectors (d) therefore formative phase needed to design an intervention. Much of this is present, but it should be distilled and signposted for readability.

5. The manuscript repeatedly contrasts public and private counselling. Consider stating upfront whether novelty lies in (i) rights-based operationalization (WHO rights framework + Theory of Change mapping), (ii) triangulation with observations, (iii) purposive inclusion of male clients, or (iv) integration into a pre-specified multi-phase intervention pathway.

3) Methods

6. Clients were selected from facility records and initially approached “through facility staff.” This can bias toward satisfied clients and inhibit critical responses. Please describe:

o how privacy was ensured during recruitment,

o whether providers/facility staff could know who participated,

o how non-response differed by facility/method type,

o and what steps reduced social desirability (neutral recruiters, off-site interviews, confidentiality reassurance).

7. You describe stratified random sampling of clients “to strengthen internal validity/reduce selection bias.” While defensible, you should explain why random selection was preferred over purposive sampling for qualitative depth, and how you ensured diversity of experiences (e.g., discontinuers, side-effect experiences, first-time vs repeat users). Also clarify how “method strata” were defined (short-acting/long-acting/permanent; modern/traditional; etc.).

8. You observed 72 sessions using a structured checklist. Please specify:

o what domains were on the checklist, whether it was adapted from an existing tool,

o how observers were trained, inter-observer consistency (if >1 observer),

o how observation data were analysed and integrated with interview themes (e.g., convergence/divergence).

9. You note that some interviews (particularly public-sector) were not audio-recorded and relied on notes/written responses. Please quantify how many were not recorded, and discuss implications (loss of nuance, differential depth by sector) and what you did to mitigate (expanded notes, immediate debrief, respondent validation).

10. The analytic paragraph is generic. The readers will expect:

• who coded (how many coders),

• whether a codebook existed and evolved,

• how disagreements were resolved,

• how saturation was assessed (and for which groups),

• how frameworks (rights-based + Theory of Change) were applied in practice (e.g., mapping themes to rights domains), and

• whether negative/deviant cases were sought.

11. Ethics section mentions adolescents aged 15–17 with guardian consent/assent; yet the sample is described as "72 married clients." Please clarify whether any 15–17-year-olds were included, how many, and whether this was anticipated in the protocol. If none were included, remove or contextualize that statement to avoid confusion.

12. You recruited 35 key informants across categories but then exclude most from the analysis. That is acceptable, but it must be reflected consistently throughout methods, abstract, and results. Also explain why excluding them strengthens the focus (or whether another paper will report them).

4) Results

13. You say, "eight core domains were explored" and then present "Theme 1, Theme 2…" Clarify whether domains were topic areas in the interview guide and themes are analytic outputs. Readers often confuse these, and clear wording will help.

14. The public/private contrast is clear and well-supported with quotes, but at times the narrative implies intent (e.g., "steering") where alternative explanations (method availability constraints, risk-aversion, workload) also exist. You can still present "steering," but you should distinguish:

• perceived steering (client perspective),

• structural steering (stock-outs/limited method mix),

• and intentional coercion (which requires stronger evidence).

15. Since you intentionally sampled 36 men and 36 women, readers will expect more explicit comparisons (where do men differ in understanding, questioning, decision-making role?). You have early signals (men's hesitation) but consider summarizing gendered patterns more systematically across themes.

16. The manuscript states triangulation across IDIs/observations/field notes, but results presented (in the visible sections) are heavily interview-quote driven. Add clear “Observation corroborated/contradicted…” statements and provide at least a few observation-derived examples.

5) Discussion & Conclusions

17. The paper acknowledges geographic limitation, but the discussion should more carefully separate: (i) what is likely system-wide in Pakistan (supported by national surveys/literature) vs (ii) what is facility-context specific (Islamabad/Rawalpindi, one private FPAP facility, two RHCs).

18. Since the work is framed around WHO rights-based counselling, the implications would be stronger if organized explicitly by rights domains (privacy, informed choice, non-discrimination, accountability) and linked to feasible facility-level and system-level actions (training, supervision, job aids, commodity security, follow-up systems).

19. You mention a subsequent cluster RCT and a multi-phase programme. Provide a concise description of the specific components this formative work will shape (e.g., counselling tool/job aid, provider training modules, follow-up mechanism, supervision checklist), to show a clear translational pathway.

20. Please ensure the manuscript includes researcher reflexivity/positionality, relationship with participants, setting/context detail, data saturation approach, and an audit trail (even briefly). Currently, these elements are not clearly described in the provided sections.

Minor comments

• Minor grammar issue in conclusion sentence (“…enhanced follow-up mechanisms is essential…” should be “are essential” or rewrite).

• Keep terminology consistent: “client-centered” vs “person-centered;” “counselling session” vs “consultation.”

• Provide exact dates without parenthetical “inclusive of travel” unless relevant to context/seasonality.

• Clarify facility names consistently (Bharakahu/Bahrakeho spelling appears inconsistent).

• Consider a small table summarizing participant characteristics by facility and gender (age bands are mentioned, but more context helps).

• Ensure quotes are formatted consistently (participant ID, facility code, gender), and reduce overly long quote strings by pairing fewer, more illustrative quotes with stronger synthesis.

---

| | |
|---|---|
| **Reviewer** | **2** |
| **Name** | **Baia, Ines** |

| | |
|---|---|
| **Affiliation** | **ProChild CoLab Against Poverty and Social Exclusion - Association** |
| **Date** | **22-Apr-2026** |
| **COI** | **None** |

I would like to thank the opportunity to review the paper "Strengthening Contraceptive Counselling Services in Pakistan: Formative evidence from a multiphase ", as it was a very interesting reading.

I would like to point out some doubts and suggestions:

- In the introduction section I thinkthe authors could provide some more context regarding family planning in Pakistan, for example if contraception is free and provided at healthcare centres; and if there are differences in the provision of this regarding public and private sectors.

- In the methods section, in the description of participants' selection for in depth interviews, I was wondering if the 72 participants were married between each other, I mean women were selected and then the corresponding husband?

- Why only "family planning clients who had used any contraceptive method during the preceding 12 months"? What about those who dont use contraceptive method?

- In my opinion both the results and discussion sections are very robust and well described.

## VERSION 1 - AUTHOR RESPONSE

| **Reviewer: 1**<br>**Dr. Shiyam Tikmani, Aga Khan University** | Thank you for the detailed feedback. |
|---|---|
| **Major comments**<br>**1) Abstract - inconsistencies**<br>1. The abstract frames the study as “mixed-methods participatory” (IDIs + observations + thematic analysis). In the manuscript, the primary analytic emphasis appears qualitative; if “mixed methods” is intended, clarify what the quantitative element is (if any), how it was analysed, and how integration occurred. Otherwise, rephrase to “multi-method qualitative” (IDIs & observations).<br>2. The abstract lists clients, providers, and 10 stakeholders; later, the methods state that broader stakeholder KIIs were collected but excluded from this paper’s | 1.Design revised to “formative, multi-method qualitative study” using IDIs, observations and thematic analysis.<br><br>2. Response: Participants section revised to state that the article analyses clients, frontline providers and observations; broader stakeholder interviews are excluded from this paper. |

| | |
|---|---|
| analysis. The abstract should explicitly state which respondent groups are included in the analysis clients + providers + observations) and which are excluded.<br>3. Statements implying national-level insufficiency are plausible but should be framed as findings from three facilities in two districts, not as definitive "Pakistan's current model" unless clearly supported by broader evidence. | 3.Conclusion and discussion rephrased to locate findings in three Islamabad/Rawalpindi facilities and discuss wider relevance as transferability rather than national generalisation. |
| **2) Introduction**<br>4. The introduction is conceptually strong (rights-based framing), but it would benefit from a more explicit logic chain: (a) discontinuation/unmet need in Pakistan (b) counselling quality affects autonomy/continuation (c) limited empirical evidence triangulating client/provider perspectives across sectors (d) therefore formative phase needed to design an intervention. Much of this is present, but it should be distilled and signposted for readability.<br>5. The manuscript repeatedly contrasts public and private counselling. Consider stating upfront whether novelty lies in (i) rights-based operationalization (WHO rights framework + Theory of Change mapping), (ii) triangulation with observations, (iii) purposive inclusion of male clients, or (iv) integration into a pre-specified multi-phase intervention pathway. | 4.A signposting paragraph was added linking unmet need/discontinuation, counselling quality, evidence gaps and need for formative intervention design.<br><br>5.A novelty paragraph has been added highlighting rights-based operationalisation, triangulation, male client inclusion and linkage to a multiphase intervention pathway |
| 3) Methods<br>6. Clients were selected from facility records and initially approached "through facility staff." This can bias toward satisfied clients and inhibit critical responses. Please describe:<br>o how privacy was ensured during recruitment,<br>o whether providers/facility staff could know who participated,<br>o how non-response differed by facility/method type,<br>o and what steps reduced social desirability (neutral recruiters, off-site interviews, confidentiality reassurance).<br>7. You describe stratified random sampling of clients "to strengthen internal | 6.Methods expanded to explain that facility staff only made initial contact, interviews were conducted by trained researchers, providers did not access responses, confidentiality was emphasised and interviews were held in private/neutral spaces where feasible.<br><br>7.Sampling section revised to define method strata and explain that random selection was used to reduce gatekeeper selection and ensure method-mix variation among recent |

| | |
|---|---|
| validity/reduce selection bias." While defensible, you should explain why random selection was preferred over purposive sampling for qualitative depth, and how you ensured diversity of experiences (e.g., discontinuers, side-effect experiences, first-time vs repeat users). Also clarify how "method strata" were defined (short-acting/long-acting/permanent; modern/traditional; etc.).<br>8. You observed 72 sessions using a structured checklist. Please specify:<br>o what domains were on the checklist, whether it was adapted from an existing tool,<br>o how observers were trained, inter-observer consistency (if >1 observer),<br>o how observation data were analysed and integrated with interview themes (e.g., convergence/divergence).<br>9. You note that some interviews (particularly public-sector) were not audio-recorded and relied on notes/written responses. Please quantify how many were not recorded, and discuss implications (loss of nuance, differential depth by sector) and what you did to mitigate (expanded notes, immediate debrief, respondent validation).<br>10. The analytic paragraph is generic. The readers will expect:<br>• who coded (how many coders),<br>• whether a codebook existed and evolved,<br>• how disagreements were resolved,<br>• how saturation was assessed (and for which groups),<br>• how frameworks (rights-based + Theory of Change) were applied in practice (e.g., mapping themes to rights domains), and<br>• whether negative/deviant cases were sought.<br>11. Ethics section mentions adolescents aged 15–17 with guardian consent/assent; yet the sample is described as "72 married clients." Please clarify whether any 15–17-year-olds were included, how many, and whether this was anticipated in the protocol. If none were included, remove or | service users. A placeholder remains for author confirmation of non-response by facility/method type.<br><br>8.Observation section revised to describe checklist domains, basis in WHO rights-based principles, observer training and integration of observation data with IDI themes.<br><br>9.Data collection section revised to describe mitigation through expanded notes, interviewer debriefing and comparison with field summaries. Placeholder added for exact number requiring author confirmation.<br><br>10.Data analysis section substantially expanded to describe two lead analysts, preliminary codebook, consensus coding, saturation, framework mapping, triangulation and negative/deviant case analysis.<br><br>11.Ethics section revised to clarify that although the wider protocol allowed adolescents, the present analysis reports married adult clients only.<br><br>12.Abstract, methods and results now consistently state that policy/donor/stakeholder interviews are excluded from this paper and reserved for separate analysis. |

| | |
|---|---|
| contextualize that statement to avoid confusion.<br>12. You recruited 35 key informants across categories but then exclude most from the analysis. That is acceptable, but it must be reflected consistently throughout methods, abstract, and results. Also explain why excluding them strengthens the focus (or whether another paper will report them). | |
| **4) Results**<br>13. You say, “eight core domains were explored” and then present “Theme 1, Theme 2…” Clarify whether domains were topic areas in the interview guide and themes are analytic outputs. Readers often confuse these, and clear wording will help.<br>14. The public/private contrast is clear and well-supported with quotes, but at times the narrative implies intent (e.g., “steering”) where alternative explanations (method availability constraints, risk-aversion, workload) also exist. You can still present “steering,” but you should distinguish:<br>• perceived steering (client perspective),<br>• structural steering (stock-outs/limited method mix),<br>• and intentional coercion (which requires stronger evidence).<br>15. Since you intentionally sampled 36 men and 36 women, readers will expect more explicit comparisons (where do men differ in understanding, questioning, decision-making role?). You have early signals (men’s hesitation) but consider summarizing gendered patterns more systematically across themes.<br>16. The manuscript states triangulation across IDIs/observations/field notes, but results presented (in the visible sections) are heavily interview-quote driven. Add clear “Observation corroborated/contradicted…” statements and provide at least a few observation-derived examples. | 13.Results introduction revised to state that eight domains were data collection topic areas, while themes are analytic outputs.<br>14.Results now distinguish perceived steering, structural steering and intentional coercion.<br>15.A gendered-patterns paragraph was added summarising differences in women’s and men’s concerns, participation and couple counselling.<br>16.Observation-based synthesis paragraphs added to show corroboration and divergence between interview accounts and observed counselling practice |
| **5) Discussion & Conclusions**<br>17. The paper acknowledges geographic limitation, but the discussion should more carefully separate: (i) what is likely system-wide in Pakistan (supported by | 17.Discussion revised to distinguish findings consistent with wider Pakistani evidence from findings specific to Islamabad/Rawalpindi and the selected facilities. |

| | |
|---|---|
| national surveys/literature) vs (ii) what is facility-context specific (Islamabad/Rawalpindi, one private FPAP facility, two RHCs).<br>18. Since the work is framed around WHO rights-based counselling, the implications would be stronger if organized explicitly by rights domains (privacy, informed choice, non-discrimination, accountability) and linked to feasible facility-level and system-level actions (training, supervision, job aids, commodity security, follow-up systems).<br>19. You mention a subsequent cluster RCT and a multi-phase programme. Provide a concise description of the specific components this formative work will shape (e.g., counselling tool/job aid, provider training modules, follow-up mechanism, supervision checklist), to show a clear translational pathway.<br>20. Please ensure the manuscript includes researcher reflexivity/positionality, relationship with participants, setting/context detail, data saturation approach, and an audit trail (even briefly). Currently, these elements are not clearly described in the provided sections. | 18.New rights-domain paragraph added, linking informed choice, privacy/confidentiality, non-discrimination/respect and accountability to facility/system actions.<br>19. Required information inserted.<br>20.In response, a new paragraph on researcher reflexivity and trustworthiness has been added. In addition, details on saturation, audit trail, debriefing, triangulation, negative/deviant cases, and thematic synthesis have also been incorporated into the Methods section. |
| **Minor comments**<br>• Minor grammar issue in conclusion sentence ("…enhanced follow-up mechanisms is essential…" should be "are essential" or rewrite).<br>• Keep terminology consistent: "client-centered" vs "person-centered;" "counselling session" vs "consultation."<br>• Provide exact dates without parenthetical "inclusive of travel" unless relevant to context/seasonality.<br>• Clarify facility names consistently (Bharakahu/Bahrakeho spelling appears inconsistent).<br>• Consider a small table summarizing participant characteristics by facility and gender (age bands are mentioned, but more context helps).<br>• Ensure quotes are formatted consistently (participant ID, facility code, gender), and reduce overly long quote strings by pairing | Agreed and responded. Conclusion grammar revised; terminology standardised to client-centred/counselling; dates simplified; Bharakahu spelling standardised; participant distribution retained and expanded; quote/context consistency flagged for final copyediting.<br>Thank you for this helpful suggestion. Rather than adding a separate table, we have expanded the participant description in the Methods section. The revised text now reports the distribution of participants by facility, gender and age group. It specifies that 24 participants were recruited from each of the three facilities, with 12 women and 12 men from each facility, and that 43 |

| | |
|---|---|
| fewer, more illustrative quotes with stronger synthesis. | participants (59.7%) were aged 19–39 years and 29 (40.3%) were aged 40 years or older. We believe this concise description provides the requested participant context while avoiding duplication in a separate table. |
| **Reviewer: 2**<br>**Ms. Ines Baia, ProChild CoLab Against Poverty and Social Exclusion - Association** | Thank you for the feedback. |
| I would like to thank the opportunity to review the paper "Strengthening Contraceptive Counselling Services in Pakistan: Formative evidence from a multiphase ", as it was a very interesting reading.<br>I would like to point out some doubts and suggestions:<br>- In the introduction section I think the authors could provide some more context regarding family planning in Pakistan, for example if contraception is free and provided at healthcare centres; and if there are differences in the provision of this regarding public and private sectors.<br>- In the methods section, in the description of participants' selection for in depth interviews, I was wondering if the 72 participants were married between each other, I mean women were selected and then the corresponding husband?<br>- Why only "family planning clients who had used any contraceptive method during the preceding 12 months"? What about those who dont use contraceptive method?<br>- In my opinion both the results and discussion sections are very robust and well described. | Introduction expanded to describe public/private FP service provision and why sector differences matter.<br><br>Sampling text clarifies 72 married clients were selected from facility records and balanced by sex/facility; it does not assume paired spouses unless explicitly recruited as such.<br><br>Methods revised to explain this focus on recent service users and acknowledge exclusion of non-users as a limitation.<br><br>Thank you! |
